# Relationship Between Serum NMDA Receptor Antibodies and Response to Antipsychotic Treatment in First-Episode Psychosis

**DOI:** 10.1016/j.biopsych.2020.11.014

**Published:** 2021-07-01

**Authors:** Thomas A. Pollak, Angela Vincent, Conrad Iyegbe, Ester Coutinho, Leslie Jacobson, Dan Rujescu, James Stone, Julie Jezequel, Veronique Rogemond, Stephane Jamain, Laurent Groc, Anthony David, Alice Egerton, Rene S. Kahn, Jerome Honnorat, Paola Dazzan, Marion Leboyer, Philip McGuire

**Affiliations:** a Department of Psychosis Studies, Institute of Psychiatry, Psychology and Neuroscience, King’s Health Partners, King’s College London, London, United Kingdom; b Division of Neuroscience, Institute of Psychiatry, Psychology and Neuroscience, King’s Health Partners, King’s College London, London, United Kingdom; c Department of Neuroimaging, Centre for Neuroimaging Sciences, Institute of Psychiatry, Psychology and Neuroscience, King’s Health Partners, King’s College London, London, United Kingdom; d Centre for Developmental Neurobiology, Institute of Psychiatry, Psychology and Neuroscience, King’s Health Partners, King’s College London, London, United Kingdom; e Department of Psychological Medicine, Institute of Psychiatry, Psychology and Neuroscience, King’s Health Partners, King’s College London, London, United Kingdom; f South London and Maudsley NHS Foundation Trust, University College London, London, United Kingdom; g Institute of Mental Health, University College London, London, United Kingdom; h Nuffield Department of Clinical Neurosciences, John Radcliffe Hospital, Oxford, United Kingdom; i Department of Psychiatry, Psychotherapy, and Psychosomatics, Martin-Luther-University Halle-Wittenberg, Halle, Germany; j Rare Disease Reference Center on Autoimmune Encephalitis, Hospices Civils de Lyon, Institut NeuroMyoGene Institut National de la Santé et de la Recherche Médicale U1217/Centre National de la Recherche Scientifique, University Claude Bernard, Universite de Lyon, Lyon, France; k Psychiatry and Addictology Department (DMU IMPACT), University Paris Est Créteil, Hopitaux Universitaires Henri Mondor, L'Assistance Publique-Hôpitaux de Paris, Créteil, France; l Translational Neuropsychiatry Laboratory, Institut National de la Santé et de la Recherche Médicale U955, Créteil, France; m FondaMental Foundation, Créteil, France; n Interdisciplinary Institute for Neuroscience, University of Bordeaux, Bordeaux, France; o Department of Psychiatry, Icahn School of Medicine at Mount Sinai, New York, New York

**Keywords:** Antipsychotics, Autoantibodies, Biomarkers, First-episode psychosis, Immunopsychiatry, NMDA receptor

## Abstract

**Background:**

When psychosis develops in NMDA receptor (NMDAR) antibody encephalitis, it usually has an acute or subacute onset, and antipsychotic treatment may be ineffective and associated with adverse effects. Serum NMDAR antibodies have been reported in a minority of patients with first-episode psychosis (FEP), but their role in psychosis onset and response to antipsychotic treatment is unclear.

**Methods:**

Sera from 387 patients with FEP (duration of psychosis <2 years, minimally or never treated with antipsychotics) undergoing initial treatment with amisulpride as part of the OPTiMiSE (Optimization of Treatment and Management of Schizophrenia in Europe) trial (ClinicalTrials.gov number NCT01248195) were tested for NMDAR IgG antibodies using a live cell–based assay. Symptom severity was assessed using the Positive and Negative Syndrome Scale and the Clinical Global Impressions Scale at baseline and again after 4 weeks of treatment with amisulpride.

**Results:**

At baseline, 15 patients were seropositive for NMDAR antibodies and 372 were seronegative. The seropositive patients had similar symptom profiles and demographic features to seronegative patients but a shorter duration of psychosis (median 1.5 vs. 4.0 months; *p* = .031). Eleven seropositive and 284 seronegative patients completed 4 weeks of amisulpride treatment: after treatment, there was no between-groups difference in improvement in Positive and Negative Syndrome Scale scores or in the frequency of adverse medication effects.

**Conclusions:**

These data suggest that in FEP, NMDAR antibody seropositivity alone is not an indication for using immunotherapy instead of antipsychotic medications. Further studies are required to establish what proportion of patients with FEP who are NMDAR antibody seropositive have coexisting cerebrospinal fluid inflammatory changes or other paraclinical evidence suggestive of a likely benefit from immunotherapy.

SEE COMMENTARY ON PAGE e1

Serum NMDA receptor (NMDAR) antibodies have been inconsistently detected in a subgroup of patients with psychosis, with the proportion varying (between 0% and 20%) with the type of assay used and the nature of the patient sample (1, 2, 3, 4). The clinical significance of NMDAR antibodies in patients with psychotic disorders remains unclear. It has been suggested that they may demarcate an autoimmune encephalitis caught early or a forme fruste of autoimmune encephalitis that has been misdiagnosed as a primary psychotic disorder. However, NMDAR antibodies are also detectable in healthy individuals, with some studies reporting similar rates to those observed in patient populations (5).

If, in a subset of patients, psychosis is caused by NMDAR antibodies, treatment with antipsychotic medication might not be effective. Case reports and series of patients with NMDAR antibody encephalitis have described a poor response to antipsychotic treatment in the initial, psychiatric phase of the disease. In addition, patients with NMDAR antibody encephalitis may be particularly sensitive to the adverse effects of antipsychotic medication, such as pronounced rigidity, rhabdomyolysis, and neuroleptic malignant syndrome (6,7). However, the effectiveness and safety of antipsychotic medication has yet to be examined in patients who have NMDAR antibodies and present with psychosis in the absence of encephalitis.

Typically, NMDAR antibody encephalitis has a subacute onset, defined as a rapid progression of symptoms of less than 3 months in duration (8). If NMDAR antibodies underlie the onset of some cases of psychosis, one might therefore expect a similarly rapid progression and, thus, shorter duration of untreated psychosis before presentation to clinical services. This hypothesis remains to be tested.

It has been suggested that immunotherapy may be indicated as an alternative to antipsychotic medication in patients with psychosis who are seropositive for NMDAR antibodies (9) (and anecdotally, we have heard of such patients being offered immunotherapy). This is partly due to observations that in the context of NMDAR antibody encephalitis, antipsychotic medications may lack efficacy in reducing psychotic symptoms, whereas immunotherapy may be effective (9,10). However, these observations remain controversial (11,12). Evaluating the safety and efficacy of antipsychotic medication in NMDAR antibody seropositive or seronegative patients with psychosis may help to resolve this issue.

The main aims of this study were to examine the relationship between serum NMDAR antibodies and 1) symptom profile at presentation and 2) response to antipsychotic treatment in a large cohort of patients with first-episode psychosis (FEP). We tested the hypothesis that, compared with seronegative patients, seropositive patients would have 1) a shorter duration of psychosis and 2) a poor therapeutic response but a greater frequency of adverse effects.

## Methods and Materials

### Subjects

We studied patients in OPTiMiSE (Optimization of Treatment and Management of Schizophrenia in Europe; www.optimisetrial.eu), a European Union–funded study of the management of FEP that recruited patients between May 26, 2011, and May 15, 2016. Full details of the trial have been reported elsewhere (13). Briefly, inclusion required that patients had a diagnosis of first-episode schizophrenia, schizoaffective disorder, or schizophreniform psychosis and were either naïve to antipsychotic medication or had received no more than 2 weeks of medication in the previous year or no more than 6 weeks in their lifetime. Inclusion also required that the time between psychosis onset and study entry was less than 2 years. Patients were excluded if there was any suspicion of an organic etiology; they had a known intolerance to one of the study drugs; they met any of the contraindications for any of the study drugs as mentioned in the (local) package insert texts; they were coercively treated or represented by a legal guardian, or both, or under legal custody; or they were pregnant or breastfeeding. Recruitment involved centers in the United Kingdom, the Netherlands, Denmark, France, Austria, Czech Republic, Germany, Israel, Italy, Poland, Spain, Australia, Bulgaria, and Romania (Table S1).

This study was restricted to patients in whom serum had been collected at presentation (*n* = 387 of 446). At baseline, subjects completed a sociodemographic schedule, and symptom severity was assessed using the Positive and Negative Syndrome Scale (PANSS) (14) and the Calgary Depression Scale for Schizophrenia (CDSS) (15). Patients were then treated with amisulpride following a standardized protocol for 4 weeks (13). The duration of untreated psychosis was estimated at baseline, using information from the patient, relatives and caregivers, and clinical records. It was defined as the time (in months) between the onset of frank psychotic symptoms to the baseline assessment. At 4 weeks, clinical response to amisulpride was assessed using the PANSS, CDSS, and the Clinical Global Impressions (CGI) Scale (16). Adverse effects of treatment were assessed using the Udvalg for Kliniske Undersogelser side effect rating scale (17). Remission was defined according to the criteria by Andreasen *et al.* (18), according to which 8 specific symptoms (PANSS items P1, P2, P3, N1, N4, N6, G5, and G9) of schizophrenia as measured by the PANSS (14) are at most only mildly present (maximum rating of 3) so that they do not interfere with daily life functioning. However, because the assessment was conducted after 4 weeks, the conventional requirement for meeting the above remission criteria for 6 months (18) was not used. All clinical ratings were completed, entered in the database, and locked before antibody testing; therefore, analyses were retrospective, and antibody status was not known at enrollment.

### Immunoassays

Serum samples were tested for NMDAR IgG antibodies using a live cell–based assay as described above (4). A live cell–based assay was used because we found that this had greater sensitivity than a fixed cell–based assay, detecting sevenfold more positive samples, which all nonetheless showed on single nanoparticle imaging a signature strongly indicative of binding to the NMDAR (4).

### Statistical Analysis

Statistical analyses were performed using SPSS (version 23; IBM Corp., Armonk, NY). Demographic variables were compared between seropositive and seronegative patients using independent samples *t* tests and Mann-Whitney *U* tests for continuous variables and χ^2^ and Fisher’s exact tests for categorical data. For clinical scales (PANSS, CDSS, and CGI), multiple linear regressions were carried out within the general linear model with NMDAR antibody status (positive or negative), sex, and race as factors and with age as covariate. Significance threshold was set to *p*  <  .05; mean ± SD are presented unless otherwise stated. Owing to the exploratory nature of the analyses, significance values are given for two-tailed tests where applicable and uncorrected for multiple comparisons.

## Results

### Participants and Prevalence of NMDAR Antibodies

A total of 387 subjects had a baseline assessment and blood sample analyzed. Fifteen of 387 (3.9%) subjects had NMDAR IgG antibodies detectable in serum, and 372 were seronegative. These subjects were included in the analyses of demographic data, duration of untreated psychosis, and baseline symptoms. Demographic and baseline clinical data are presented in Table 1.

**Table 1:** Demographic and Basic Clinical Information at Baseline

Demographic and Clinical Data	Total FEP Cohort, *n* = 387	NMDAR Ab Seronegative, *n* = 373	NMDAR Ab Seropositive, *n* = 15	*p* Value, Seronegative vs. Seropositive
Age, Years	25.44 ± 6.10	26.00 ± 6.58	25.42 ± 6.08	.719
Sex, Male	261 (67.4%)	253 (68.0%)	8 (53.3%)	.265
Race	White 346 (89.6%), Black 16 (4.1%), Asian 13 (3.4%), other 11 (2.9%)	White 332 (89.5%), Black 15 (4.0%), Asian 13 (3.5%), other 11 (3.0%)	White 14 (93.3%), Black 1 (6.3%), Asian 0 (0.0%), other 0 (0.0%)	.745
Current Smoker, *n* = 385	192 (49.9%)	184 (49.6%)	8 (57.1%)	.391
Body Mass Index	23.41 ± 5.20	23.42 ± 5.24	23.19 ± 4.09	.867
Recreational Drug Use Ever, *n* = 386	184 (47.7%)	177 (47.7%)	7 (46.7%)	1.000
Duration of Psychosis, Mo	6.05 (4.00) [2–8]	6.13 (4.00) [2–8]	4.00 (1.5) [1–3.5]	.031^a^

Values are presented as mean ± SD, *n* (%), or mean (median) [interquartile range].

Ab, antibody; FEP, first-episode psychosis; NMDAR, NMDA receptor.

a Mann-Whitney *U* test.

In total, 92 subjects were excluded from the follow-up analysis. Two subjects dropped out of the study before receiving amisulpride (one because of returning to their home country to be with family, another because of moving to a different region of the same country); both were NMDAR antibody seronegative. A further 90 subjects received amisulpride but were excluded (either dropped out/excluded before finishing 4 weeks of treatment or excluded from analyses after completion of treatment) because of the following reasons: adverse events (*n* = 8), protocol violation (*n* = 42), subject did not wish to continue (*n* = 27), subject died (*n* = 1), at the discretion of the investigator (*n* = 4; includes violence [*n* = 1], prolonged hospitalization [*n* = 1], clinical inefficacy [*n* = 1], retrospectively felt not to meet criteria [*n* = 1]) and other reasons (*n* = 8). Of these 90 excluded subjects, 4 were seropositive for NMDAR antibodies. Reasons for exclusion of these 4 patients were protocol violation (*n* = 1), death (died by suicide) (*n* = 1), withdrew because of weight gain and fatigue (*n* = 1), and other (lost to follow-up: *n* = 1).

Table S1 summarizes demographic and clinical differences between subjects who were included and excluded (dropped out or excluded after treatment as described above) for follow-up. The frequency of NMDAR antibodies was 3.7% in the subjects who were included in the follow-up study and 4.3% in subjects excluded from the follow-up study (*p* = .761). Excluded subjects were less frequently white than included subjects. The duration of psychosis was shorter for excluded (median, 3 months) than for included (median, 4 months) subjects (*p* = .041). In total, 295 (11 seropositive and 284 seronegative) subjects completed 4 weeks of amisulpride treatment; these subjects were included in analyses of treatment response.

**Figure 1:**
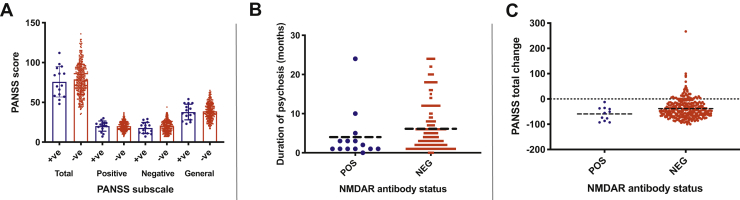
**(A)** Positive and Negative Syndrome Scale (PANSS) subscale scores by serostatus. **(B)** Duration of psychosis at baseline. **(C)** Percentage change in PANSS score after 4 weeks of amisulpride treatment, by serostatus. Neg, negative; NMDAR, NMDA receptor; Pos, positive.

The remaining seropositive subjects did not differ from those who were seronegative with respect to age, gender, body mass index, smoking status, or recreational drug use (Table 1). However, the duration of untreated psychosis was significantly shorter in the seropositive subjects: the median duration was 1.5 months (interquartile range, 1–3.5), compared with 4.0 months (interquartile range, 2–8) in the seronegative subjects (*p* = .031) (Figure 1B).

### Clinical Associations of NMDAR Antibody Seropositivity

At baseline, subjects who were NMDAR antibody seropositive did not differ from seronegative subjects on any measure of symptom severity (Table 2 and Figure 1).

**Table 2:** Psychopathology and Functioning Scores by Serostatus at Baseline

Baseline Measure	NMDAR Ab Negative, *n* = 373	NMDAR Ab Positive, *n* = 15	B^a^	*p* Value, NMDAR Ab Negative vs. Positive
PANSS Total	78.78 ± 19.00	75.80 ± 19.10	3.27	.513
PANSS Positive	20.12 ± 5.55	20.13 ± 6.38	0.096	.949
PANSS Negative	19.89 ± 7.10	17.80 ± 6.95	1.92	.302
PANSS General	38.76 ± 9.99	37.87 ± 9.86	1.26	.631
CDSS	13.31 ± 4.48	13.07 ± 4.37	0.362	.759
CGI	5.53 ± 0.92	5.53 ± 1.19	−0.001	.998

Continuous variables are presented as mean ± SD.

Ab, antibody; CDSS, Calgary Depression Scale for Schizophrenia; CGI, Clinical Global Impressions; NMDAR, NMDA receptor; PANSS, Positive and Negative Syndrome Scale.

a Regression coefficient from multiple regression procedure.

### Response to Antipsychotic Treatment

The mean reduction in PANSS total score over the treatment period was not greater in subjects who were seropositive than in those who were seronegative (*p* = .098) (Figure 1C), nor was the CGI score at 4 weeks different between groups (*p* = .075). CGI improvement scores at 4 weeks indicated a greater degree of observed clinical improvement relative to baseline in seropositive patients (2.64 ± 0.67) than in seronegative patients (3.34 ± 1.04; *p* = .044) (Table 3).

**Table 3:** Measures of Clinical Response by Serostatus After 4 Weeks Amisulpride Treatment

Measure	NMDAR Ab Negative, *n* = 284	NMDAR Ab Positive, *n* = 11	B^a^	*p* Value, NMDAR Ab Negative vs. Positive
PANSS Total, Baseline	78.89 ± 19.28	74.21 ± 18.77	5.04	.341
PANSS Total, Posttreatment	58.94 ± 18.32	50.45 ± 20.46	8.22	.145
Percentage Change in Total PANSS Scores	−38.01 ± 38.97	−59.56 ± 26.91	19.55	.098
Percentage Change in CDSS Scores	−7.25 ± 24.81	−10.54 ± 12.31	2.64	.741
CGI, Posttreatment	4.41 ± 1.09	3.82 ± 1.25	0.604	.075
CGI Improvement	3.34 ± 1.04	2.64 ± 0.67	4.27	.044
Number in Remission	196 (69.3%)	9 (81.8%)	0.683	.394

Values are presented as mean ± SD or *n* (%).

Ab, antibody; CDSS, Calgary Depression Scale for Schizophrenia; CGI, Clinical Global Impressions; NMDAR, NMDA receptor; PANSS, Positive and Negative Syndrome Scale.

a Regression coefficient from multiple or logistic regression procedure.

There was no association between frequency of remission and serostatus, with remission occurring in 196 (69.3%) seronegative patients and 9 (81.8%) seropositive patients (*p* = .394). There was no difference between seronegative and seropositive subjects in the frequency of adverse effects after 4 weeks of treatment (Table 4).

**Table 4:** Frequency of Subjects Experiencing Adverse Events

Type of Adverse Event	NMDAR Ab Negative, *n* = 284	NMDAR Ab Positive, *n* = 11	*p* Value, NMDAR Ab Negative vs. Positive
Psychic	144 (50.7%)	7 (63.6%)	.542
Neurological	55 (19.4%)	3 (27.3%)	.457
Autonomic	69 (23.3%)	4 (36.4%)	.474
Other	119 (41.9%)	8 (72.7%)	.061
None	60 (21.1%)	2 (18.2%)	1.000

Values are presented as *n* (%).

Ab, antibody; NMDAR, NMDA receptor.

## Discussion

Our first hypothesis was that seropositive patients with FEP would have a relatively short duration of untreated psychosis on the basis that the presentation of NMDAR antibody encephalitis is typically subacute. This hypothesis was supported: seropositive subjects had a significantly shorter duration of psychosis before the baseline assessment than seronegative subjects. This difference was not attributable to serostatus-associated differences in variables that have been (inconsistently) associated with a shorter duration of untreated psychosis, such as male sex, younger age at diagnosis, or substance use (19,20). Because the assessment of untreated psychosis duration was retrospective, it was difficult to precisely measure when frank psychotic symptoms first emerged. This issue could be addressed by conducting a prospective study, with ascertainment of subjects in the clinical high risk phase. However, this would require a large number of samples, as the prevalence of NMDAR antibodies in this population is similarly low [approximately 5% (21)], and only a minority of subjects with clinical high risk will later develop psychosis (22).

Our second hypothesis, partly based on data from patients with psychosis in the context of NMDAR antibody encephalitis, was that seropositive patients with FEP would show a relatively poor symptomatic response to antipsychotic medication. However, seropositive patients showed a similar improvement in symptoms to seronegative patients after 4 weeks of treatment with amisulpride, with 82% of seropositive patients achieving remission within this short time frame. This relatively good response was evident both in the investigator-rated change in the PANSS total score and in the evaluations made using the CGI, in which the mean scores of seropositive patients indicated that they were much improved, whereas mean scores of seronegative patients indicated minimal improvement in this group. Both sets of ratings were made before determining patient antibody status. Whereas the CGI improvement scale suggests a more positive response to antipsychotics in seropositive patient, the more detailed and objective PANSS failed to show such between-group differences (although the size of the seropositive group may not have been large enough to clearly demonstrate that seropositive patients do not respond better to amisulpride than seronegative patients).

We also predicted that seropositive patients would be more likely to experience adverse effects of antipsychotic medication than seronegative patients. While there was no difference in the frequency of adverse effects between the groups, this lack of difference should perhaps be interpreted with caution because of the relatively low number of seropositive patients and low frequency of adverse events. The absence of the predicted increased frequency of adverse effects in the seropositive patients was not attributable to their receiving antipsychotic drugs that are rarely associated with adverse effects or that were prescribed at low doses, as both groups were treated with the same medication in similar doses. Moreover, compared with other antipsychotics, amisulpride is relatively frequently associated with extrapyramidal side effects, which are the type that have been associated with antipsychotic treatment in NMDAR antibody encephalitis (6,7).

One patient who was seropositive for NMDAR antibodies committed suicide 7 days after discontinuing amisulpride; this was the only suicide reported. Suicidality and completed suicide have been reported in NMDAR antibody encephalitis (23) but to our knowledge have not been reported as a feature of patients without encephalitis who are seropositive for NMDAR antibodies. Post hoc analyses (not reported here) showed no difference between seropositive and seronegative FEP on the suicidality subscale of the CDSS. This finding and the observed overall lack of association between serostatus and symptom severity across domains suggest that the seropositive status in this patient was coincidental.

Our results suggest that serum NMDAR antibodies may have limited disease relevance in FEP, insofar as they were associated with a shorter duration of untreated psychosis but not with a differential response to initial antipsychotic treatment. Contrary to our hypothesis, the data suggest that NMDAR antibodies may be associated with a good response to antipsychotic treatment. We consider the most likely reason for this finding to be that NMDAR antibodies were not causative of these patients’ psychosis and indeed that they may be unrelated to their illness altogether. However, since we included no explicit measurement of central nervous system (CNS) inflammation, except by magnetic resonance imaging, which is known not to be sensitive to autoimmune encephalopathies, the possibility of some causal pathogenic role cannot be ruled out. In such an instance, which we consider unlikely, it is possible that improvements in symptoms may have been driven by factors other than antipsychotic medication in the seropositive group. In some patients with FEP, and particularly in those with a brief duration of psychosis, symptoms can remit in the absence of antipsychotic medication (24). It is also possible that psychosis associated with NMDAR antibodies is more likely to spontaneously remit (independent of treatment) than a psychotic disorder that is not associated with autoantibodies [possibly akin to other transient autoimmune phenomena such as self-limiting arthritis with evidence of autoimmune involvement (25)]. The clinical course of FEP is heterogeneous, with some patients experiencing only one episode and others exhibiting a multiepisode course or a relapsing-remitting course with accumulation of disability and comorbidity. Future work is planned to establish whether patients with FEP who are NMDAR antibody seropositive demonstrate a particular illness trajectory.

It will also be of interest to explore the longitudinal course of NMDAR antibody serostatus in patients who were seropositive (and indeed seronegative) at baseline, with a view to assessing if serostatus changes in association with symptomatic response to treatment, functional status, or relapse. There is recent evidence from humans and animals that serostatus fluctuates over time and that NMDAR antibody production may occur as a response to chronic stress (although this effect was more marked with non-IgG isotypes) (26). Furthermore, the presence of functional unmutated IgG antibodies (27) suggests the possibility that these antibodies may constitute part of the natural antibody repertoire, potentially serving an adaptive physiological function. One hypothesis might be that NMDAR antibodies in patients with psychosis are a secondary immune response to whichever nonautoimmune NMDAR dysfunction is the primary driver of the illness.

To the best of our knowledge, this is the largest study of NMDAR antibodies in FEP. However, the prevalence and, therefore, sample size of patients who are seropositive are low. Our study excluded patients with organic psychoses and required patients to be well enough to consent to and complete a clinical trial. This suggests the possibility that our sample may be biased toward patients who are more likely to show a good response to antipsychotic medication than the general clinical population. A recent study including acutely unwell patients with FEP, of whom many were incapacitous (i.e., lacked capacity to consent at the time of enrollment and blood sampling), found a somewhat higher rate of NMDAR antibody seropositivity, along with considerable evidence that the antibodies were clinically relevant in these patients (concurrent CNS NMDAR antibodies, inflammation, and good response to immunotherapy) (10).

Owing to limited serum availability, only IgG NMDAR antibodies were measured. Although IgA and IgM antibodies are not always thought to be pathogenic and indeed are not considered to be causal in NMDAR antibody encephalitis, it is conceivable that they could still have a useful role as biomarkers of antipsychotic response. Another limitation was that cerebrospinal fluid was not available for analysis, which can provide evidence of CNS inflammation by means of white blood cell count, protein count, and the presence of oligoclonal bands. A goal for future studies is to combine the measurement of serum antibodies with the collection of cerebrospinal fluid so that the prevalence of CNS inflammation in seropositive patients and its impact on clinical outcomes can be examined.

### Conclusions

This study suggests that in a patient with psychosis, a positive serum NMDAR antibody result does not indicate that treatment with antipsychotic medication will be ineffective or associated with increased adverse effects. This supports recent guidelines for the investigation and management of suspected autoimmune psychosis (28) that propose that patients who are psychotic and are identified as NMDAR antibody seropositive should undergo further electroencephalogram, magnetic resonance imaging, and cerebrospinal fluid analysis to clarify the clinical significance of the positive antibody result. Only in the presence of abnormalities confirmed in these modalities is it considered likely that NMDAR antibody seropositivity is truly indicative of active CNS inflammation and that immunotherapy might be indicated (28).

## Supplementary Material

Supplementary Material

Key Resources Table
