# Erratum

**DOI:** 10.1016/j.biopsych.2021.04.001

**Published:** 2021-07-01

Erratum to: “Relationship Between Serum NMDA Receptor Antibodies and Response to Antipsychotic Treatment in First-Episode Psychosis,” by Pollak *et al.* (*Biol Psychiatry* 2021; 90:9-15); https://doi.org/10.1016/j.biopsych.2020.11.014.

An astute reader noticed an error in the final paragraph of the Results section for this paper. Specifically, the serostatus terms were inadvertently reversed in the following sentence: “There was no association between frequency of remission and serostatus, with remission occurring in 196 (69.3%) seropositive patients and 9 (81.8%) seronegative patients (*p* = .394).” The corrected sentence reads: “There was no association between frequency of remission and serostatus, with remission occurring in 196 (69.3%) seronegative patients and 9 (81.8%) seropositive patients (*p* = .394).” The authors emphasize that the difference remains nonsignificant with no change to the significance value and the results are not influenced by this typographical error in any way.

This error has been corrected in the final paginated version of this article.

