# Supplementary material for: Relationship Between Serum NMDA Receptor Antibodies and Response to Antipsychotic Treatment in First-Episode Psychosis
Source: Biol Psychiatry. 2021 Jul 1;90(1):9–15. doi: 10.1016/j.biopsych.2020.11.014 (PMC8191702; doi:10.1016/j.biopsych.2020.11.014)
Supplement: Supplementary Material [file mmc1.pdf]

Relationship Between Serum NMDA Receptor Antibodies and Response to Antipsychotic Treatment in First-Episode Psychosis

Supplement

Table S1: Baseline demographic and clinical information in subjects who were included and excluded from analysis of amisulpride treatment response. Continuous variables given as mean ± SD unless otherwise indicated.

	**Total FEP cohort (n=387)**	**Included (n=295)**	**Excluded (n=92)**	**P (included vs excluded)**
**Age**	25.44 ± 6.10	25.48 ± 5.91	25.33 ± 6.70	0.831
**Sex [M: n (%)]**	261 (67.4)	253 (68.0)	8 (53.3)	0.265
**Ethnicity [n (%)]**	White 346 (89.6), Black 16 (4.1), Asian 13 (3.4), Other 11 (2.9)	White 271 (91.9), Black 8 (2.7), Asian 9 (3.1), Other 7 (2.4)	White 75 (82.4), Black 8 (8.8), Asian 4 (4.4), Other 4 (4.4)	**0.041**
**Smoker? [n (%)] N=385**	192 (49.9)	150 (50.8)	42 (46.7)	0.786
**BMI**	23.41 ± 5.20	23.34 ± 4.21	23.68 ± 7.63	0.688
**Recreational drug use ever [n (%)] N=386**	184 (47.7)	136 (46.4)	47 (51.6)	0.403
**Duration of psychosis (months)**	6.05 (median 4.0; IQR 2-8)	6.52 (median 4.0; IQR 2-8)	4.57 (median 3.0; IQR 1-6)	**0.041**
**NMDAR antibodies**	15 (3.9%)	11 (3.7%)	4 (4.3%)	0.761
**PANSS total (baseline)**	78.66 ± 18.99	78.39 ± 19.11	79.55 ± 18.64	0.610
**PANSS positive (baseline)**	20.12 ± 5.58	20.02 ± 5.63	20.46 ± 5.42	0.510
**PANSS negative (baseline)**	19.81 ± 7.10	19.82 ± 7.14	19.77 ± 7.00	0.955
**PANSS general (baseline)**	38.73 ± 9.98	38.55 ± 9.82	39.32 ± 10.51	0.521
**CDSS (baseline)**	13.31 ± 4.48	13.39 ± 4.54	13.01 ± 4.23	0.489
**CGI (baseline)**	5.53 ± 0.93	5.50 ± 0.94	5.64 ± 0.92	0.204

Table S2: number of participants by country and site. Individual centres listed below.

Country	**Total FEP cohort (n = 387)**	**Included in analysis of treatment response (n = 295)**
Australia	8	5
Austria	2	2
Belgium	2	2
Bulgaria	3	3
Czech Republic	19	17
Denmark	42	30
France	1	0
Germany	27	20
Israel	66	50
Italy	38	28
Netherlands	10	8
Poland	31	29
Romania	22	19
Spain	86	58
Switzerland	10	6
United Kingdom	20	18

Australia
Orygen Youth Health, 35 Poplar Rd, Parkville VIC 3052, Australia

Austria
Department of Biological Psychiatry, Innsbruck University Clinics, Innsbruck, Austria, A-6020

Belgium
Katholieke Universiteit Leuven (KU Leuven), Leuven, Belgium, B – 3070

Bulgaria
University Specialised Hospital for Active Treatment in Neurology and Psychiatry "St. Naum", Sofia, Bulgaria, 1113

Czech Republic
Psychiatrické centrum Praha, Prague, Ustavni 91, Czech Republic, 181 03 Praha 8-Bohnice

Psychiatrická klinika LF UK, Fakultní nemocnice, Hradec Králové, Czech Republic, CZ - 500 05

Denmark
Center for Neuropsychiatric Research, Glostrup, Denmark, DK-2600

France
Institut National de la Santé et de la Reserche Médicale (INSERM), Créteil Cedex, France, 94010

Germany
Martin-Luther-University (MLU) of Halle-Wittenberg, Halle, Germany, 06097

Ludwig-Maximilians University München, München, Germany, 80336

Technische Universität München (TUM), München, Germany, 81675

Israel
Sheba Medical Centre Department of Psychiatry, Tel Hashomer, Israel, 52621

Italy
Department of Psychiatry University of Naples, Naples, Italy, 80138

Netherlands
University Medical Center Utrecht
Utrecht, Netherlands, 3584 CX

Poland
Department of Adult Psychiatry, University of Medical Sciences, Poznan, Poland, 60-572

Romania
Obregia Psychiatric Hospital, Bucuresti, Romania, 7000

Spain
Hospital Clinic i Provincial, Barcelona, Spain, 08036 Barcelona

Servicio Madrileño de Salud (SERMAS), Madrid, Spain, 28007

Hospital Clínico San Carlos, Madrid, Spain, 28040 Madrid

Instituto de Investigación Hospital 12 de Octubre, Madrid, Spain, 28041 Madrid

Universidad de Oviedo, Oviedo, Spain, 33011 Oviedo

Switzerland
Clienia Schlössli AG, Privatklinik für Psychiatrie und Psychotherapie, Oetwil am See/Zürich, Switzerland, CH-8618

United Kingdom
King's College London, Departments of Psychological Medicine, Psychiatry & Cognitive Neuroscience, London, United Kingdom, SE5 8AF

West London Mental Health Trust, London, United Kingdom, W12 0NN

University of Manchester, Manchester, United Kingdom, M13 9PL
